# Prognostic impact of disease-related complications in asymptomatic mitral regurgitation: a health insurance claims analysis

**DOI:** 10.1007/s00392-024-02532-0

**Published:** 2024-08-28

**Authors:** L. Acar, C. A. Behrendt, S. Baldus, V. Falk, N. Smetak, M. Mboulla Nzomo, U. Marschall, E. Girdauskas

**Affiliations:** 1 BARMER, Wuppertal, Germany; 2 Department of Vascular and Endovascular Surgery, Asklepios Clinic Wandsbek, Asklepios Medical School, Hamburg, Germany; 3 Department III of Internal Medicine, University Hospital of Cologne, Cologne, Germany; 4 Department of Cardiothoracic and Vascular Surgery, Deutsches Herzzentrum Der Charité, DZHK Partner Site Berlin, Berlin, Germany; 5 Bundesverband Niedergelassener Kardiologen, Munich, Germany; 6 Department of Cardiothoracic Surgery, University Hospital Augsburg, Stenglinstr. 2, 86156 AugsburgAugsburg, Germany

**Keywords:** Mitral valve insufficiency, Mitral regurgitation, Cardiovascular diseases, Asymptomatic valvular heart disease, Mitral valve surgery

## Abstract

**Background and aims:**

The impact of mitral regurgitation (MR) in asymptomatic patients is not well defined. We aimed to determine the prevalence of MR-related complications and their association with 10-year survival in a large unselected asymptomatic MR cohort.

**Methods:**

Health insurance claims data from Germany’s second largest health insurance fund, BARMER, which maintains longitudinal data on 8.7 million German residents, were retrospectively analyzed. All patients with an outpatient diagnosis of MR in a minimum of two quarters during a calendar year and first recorded diagnosis between 2008 and 2011 were included. Patients with any complication attributable to MR or mitral valve intervention at index were excluded. Outcomes were compared between study group and age- and sex-matched controls (i.e., without known cardiac disease). MR-related complications of interest were new congestive heart failure, new-onset atrial fibrillation, pulmonary hypertension, or cardiac decompensation.

**Results:**

A total of 56,577 individuals (median age 68 years, 67% female) with asymptomatic MR were identified. At 10 years, MR-related complications were more frequent in the study group vs. control group (46.5% vs. 20.8%, OR 3.31, P < 0.0001). Furthermore, MR-related complications were more common in male vs. female patients with an asymptomatic MR (OR 2.65, P < 0.0001). The occurrence of at least one MR-related complication was associated with a reduced 10-year survival (OR 1.80, P < 0.0001).

**Conclusions:**

Almost half of patients with asymptomatic MR experience complications during a 10 year follow-up which result in impaired survival. These results imply the necessity of long-term disease management program. Furthermore, decision-making process and timing for mitral valve intervention in asymptomatic patients should be reevaluated.

**Graphical Abstract:**

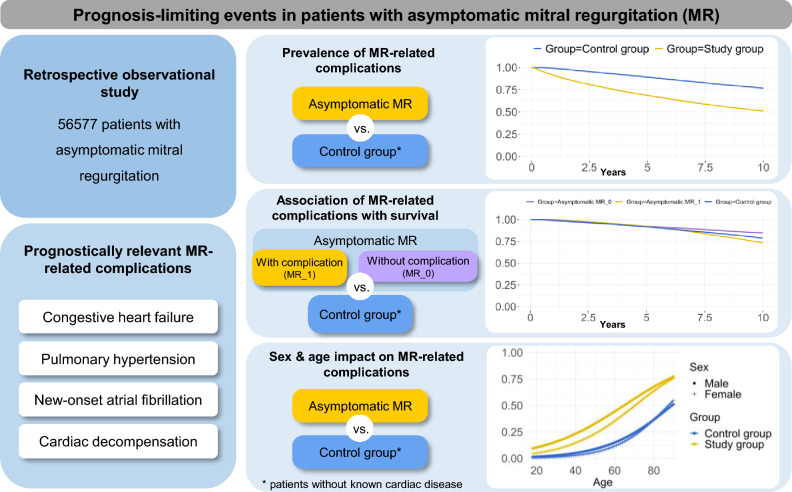

**Supplementary Information:**

The online version contains supplementary material available at 10.1007/s00392-024-02532-0.

## Introduction

Mitral regurgitation (MR) is the most common valvular heart disease (VHD) in adults [1]. Over 18 million cases of degenerative MR have been estimated globally, whereas the prevalence nearly doubled over the last 2 decades [1]. Furthermore, a post hoc analysis of OxValve Population Cohort Study revealed a 2.3% likelihood of undiagnosed moderate or severe MR, especially in the older population and in lower socioeconomic classes [2].

MR, in particular severe MR is associated with increased morbidity and mortality in all age groups and is highly amenable to surgical intervention [3, 4]. As a consequence, the 2021 ESC/EACTS guidelines for the treatment of VHD provide a Class I recommendation for surgical repair in asymptomatic patients with degenerative MR and left ventricular (LV) dysfunction [5]. The potential impact of atrial and ventricular remodeling, the development of pulmonary hypertension or atrial fibrillation in the absence of overt systolic LV dysfunction is still a matter of debate. From a pathophysiological point of view, these markers indicate an advanced activation of adaptive mechanisms, that have been recently shown to correlate with worse long-term post-surgical survival (6). Moreover, the presence of more than one of these markers is independently associated with mortality and, therefore, seems to indicate a high-risk subgroup [6]. Similar, Goliasch et al. demonstrated in heart failure patients that the prognostic impact of severe functional MR is given in a sub-cohort of *intermediate-failure phenotype*- defined by NYHA class II or III, moderate LV dysfunction and moderately increased NT-proBNP values [7]. Once the transition to a full-blown heart failure has been completed, the prognostic impact of severe functional MR is abolished.

Summarizing the above mentioned, an earlier surgery for severe MR could be reasonable, before prognostically relevant events occur. However, the risk of such events in asymptomatic MR is unknown. Therefore, the aim of this study was: (i) to determine the prevalence of MR-related complications in a large asymptomatic MR cohort in comparison to age- and sex-matched control group, and (ii) to evaluate the association between MR-related complications and 10 year survival.

## Methods

### Database

The present study was a retrospective observational study of health insurance claims data from Germany’s second largest health insurance fund BARMER, which maintains longitudinal data on outpatient and inpatient medical services for a population of up to 8.7 million German residents (10% of the German population). Diagnoses were identified using the German Modification of the International Classification of Diseases (ICD-10-GM) coding system, and procedures were identified using the Operations and Procedures Codes (OPS) coding system. The BARMER cohort comprised nationally representative data with a sex and age distribution comparable to that in Western European countries and has already been used in numerous research projects [8–10]. The Medical Service of the Health Funds in Germany routinely conducts systematic random sampling to evaluate internal and external validity. Multiple peer-reviewed validation studies have previously been documented [11–13]. In accordance with the Strengthening the Reporting of Observational Studies in Epidemiology (STROBE) guidelines, our methodological approach was meticulously structured and implemented [14].

### Study design

The inclusion criteria required an outpatient diagnosis of MR (I34.0) irrespective of classification (i.e., functional or degenerative). Moreover, an outpatient physician should record the MR diagnosis in at least two different quarters within the same calendar year. This stipulation guaranteed the consistency of the MR diagnosis over time, thereby strengthening the reliability of the study cohort. To ensure a follow-up of 10 years, the first recorded diagnosis between 2008 and 2011 was selected. Incident cases were those without a MR diagnosis coded within the preceding 2 years. It is notable that in the German outpatient sector, alongside the relevant ICD code, each diagnosis must be assigned an additional indicator for diagnostic certainty, ensuring comprehensive documentation. In all analyses of outpatient billing data, only diagnoses with the additional indicator 'confirmed' for diagnostic certainty, denoted by the diagnostic code suffix 'G', were utilized. All individuals under 18 years and older than 90 years were excluded (n = 1462). Moreover, individuals were excluded if they had at least one of the following complications attributable to MR (n = 35,443): congestive heart failure (I50-), atrial fibrillation (I48-), pulmonary hypertension (I27.2-) or cardiac decompensation (I51.9)—during the same quarter as the incidence quarter of MR (i.e., only asymptomatic MR patients were selected). Similarly, individuals were excluded if a mitral valve intervention was documented for the same quarter during which the index diagnosis was made (n = 182). Individuals were also excluded if they had no health insurance coverage 2 years before the first diagnosis (n = 3134).

A propensity score (PS)-matched control group of individuals without known cardiac disease from the entire BARMER cohort (i.e., excluding all those with diagnosis of I00–I02, I05–I09, I20–I25, I26–I28, I30–I52, or I34.0) between 2009 and 2011 was used for between-group comparison (i.e., control group). The start date for observing the control cohort was set at June 15, 2011.

### Follow-up and study endpoints

Follow-up period started with the first documentation of MR and lasted for 10-years. Participants with loss to follow-up, due to health insurance plan changes, for example, were excluded from the analysis. The study's primary endpoints were a) the prevalence of MR-related complications during a 10-year follow-up period after the initial MR diagnosis quarter and b) the impact of these complications on all-cause mortality. MR-related complications were defined as congestive heart failure (I50-), new-onset atrial fibrillation (I48-), pulmonary hypertension (I27.2-), or cardiac decompensation (I51.9).

Secondary endpoints were MR-related interventions and unplanned hospital admissions during the 10-year follow-up period. Hospital admissions were included if MR related only, i.e., MR was coded as either a primary or secondary diagnosis in combination with at least one OPS code of mitral valve intervention (Supplemental Table S1).

### Statistical analysis

Categorical variables were presented using numeric and percentage measures, while median and interquartile range (IQR) were used to describe continuous variables. To reduce potential biases in the observational data and ensure comparability with the healthy controls, we implemented propensity score matching. Age and sex were used as variables for PS modeling. Sex was matched in a 1:1 ratio, while age was matched using nearest neighbor matching. After PS-matching, there was no difference in the age and sex distribution between the both groups (Supplemental Table S2). The probabilities of remaining free from MR-related complications were compared between the study and control cohorts using the Kaplan–Meier method. Logistic regression models were used to investigate the impact of age and sex on the likelihood of experiencing MR-related complications. Survival analysis between groups was performed using the Kaplan–Meier method, with group comparisons performed by log-rank test. Two-sided tests were used to determine statistical significance, where a P-value < 0.05 denoted significance. Multiple comparisons were conducted, and a Bonferroni correction was applied to adjust for the increased risk of type I errors. The adjusted significance level was set at 0.05/N, where N is the number of tests. A Cox Proportional Hazard Regression analysis was conducted to investigate the influence of MR-related complications on 10-year survival. The analysis controlled for sex and age, in order to eliminate the potential confounding effects of these variables. Statistical analyses were performed using R, version 4.2.1.

## Results

### Asymptomatic MR cohort

Between 2008 and 2011, a total of 90,245 insured individuals (median age 70 years (IQR 62–77), 64.2% female) with a newly diagnosed MR were identified from the BARMER database, representing about 1% of the BARMER insured population (i.e., 8.7 million German residents). A higher incidence of MR was observed in females compared to males, with 132.4 vs. 113.3 individuals per 10,000 insured insurance holders (95%CI: 0.0018–0.0021, P < 0.0001). Of these individuals, 56,577 (62.7%) met the criteria for asymptomatic MR and served as our study population. Females demonstrated a significantly higher incidence of asymptomatic MR compared to males, with 87.3 vs. 64.4 individuals per 10,000 insured individuals (95%CI: 0.0022–0.0024, P < 0.0001).

### Baseline characteristics

After propensity score matching, both cohorts (i.e., asymptomatic MR cohort and control group) consisted each of 56,577 individuals and were used for all following analyses. The median age was 68 years (IQR 58–74) in both cohorts and the sex distribution was identical (i.e., 38,188 (67%) females and 18,389 (33%) males). The median follow-up duration for the cohorts was 9.14 years.

The most common comorbidities were comparable in both cohorts: arterial hypertension (I10.-), dyslipidemia (E78.-), accommodation and refraction disorders (H52.-), back pain (M54.-), climacteric disorders (N95.-), spondylosis (M47-), gonarthrosis (M17-), depressive episode (F32-), non-insulin-dependent diabetes mellitus (E11-), other non-toxic goiter (E04-), varicose veins of the lower extremities (I83-), other diseases of the spine and back (M53-), somatoform disorders (F45-), and obesity (E66-). These comorbidities in the study group as compared to the control group are summarized in the Supplemental Table S3.

### Cumulative 10-year prevalence of MR-related complications

**Fig. 1:**
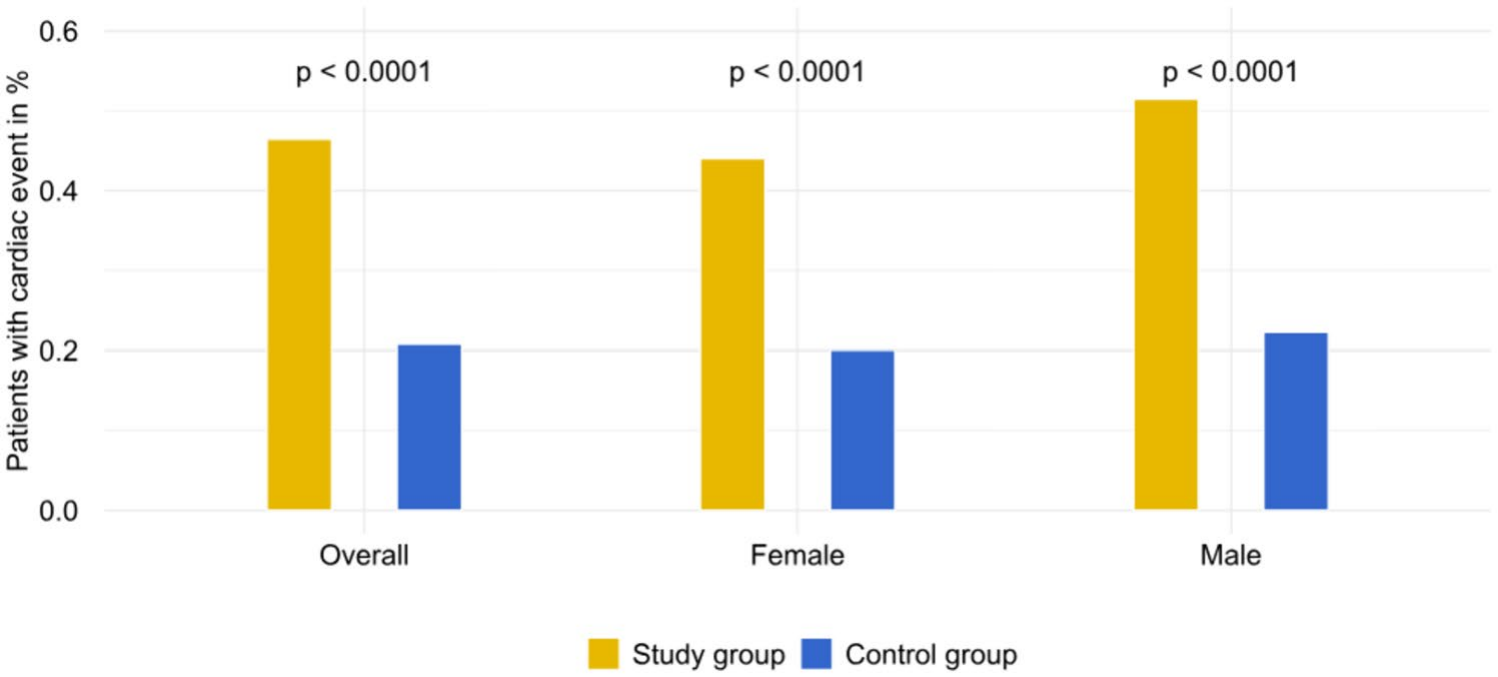
Proportion of patients with at least one MR-related complication during ten-year follow-up

The prevalence of MR-related complications was significantly different between the study and the control group. In the study group, 26,282 individuals (46.5%) developed at least one MR related complication during a 10-year follow-up period, while 11,763 individuals (20.8%) had a complication in the control group (OR: 3.31, 95%CI: 0.251–0.262, P < 0.0001) (Fig. 1). The prevalence of MR-related complications in male patients was 51.5% (n = 9463) in the study group vs. 22.3% (n = 4097) in the control group (OR: 3.70, 95%CI: 0.282–0.301, P < 0.0001). For females, the prevalence was 44.0% (n = 16,819) in the study group vs. 20.1% (n = 7666) in the control group (OR: 3.13, 95%CI: 0.233–0.246, P < 0.0001).

**Fig. 2:**
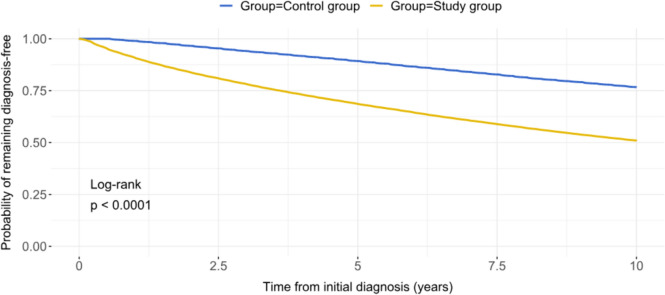
Cumulative prevalence of MR-related complications in the asymptomatic MR group vs. control group

Only one half of asymptomatic MR patients had no MR-related complications during the 10-year follow-up, as compared to 77% in the control group (P_log-rank_ < 0.0001, Fig. 2).

### Ten-year prevalence of specific MR-related complications

All four analyzed MR-related complications (i.e., new congestive heart failure, new onset atrial fibrillation, pulmonary hypertension and cardiac decompensation) occurred more frequently in the study group vs. control group, as summarized in the Supplemental Figures S1A-D. The most important difference between the groups was found in 10-year prevalence of new congestive heart failure—17,921 (31.7%) in the study group vs. 7412 (13.1%) in the control group (OR: 3.08, 95%CI: 0.181–0.191, P < 0.0001) and in the occurrence of new-onset atrial fibrillation—13,816 (24.4%) in the study group vs. 5866 (10.4%) in the control group (OR: 2.79, 95%CI: 0.136–0.145, P < 0.0001).

### Age- and sex-specific risk of MR-related complications

**Fig. 3:**
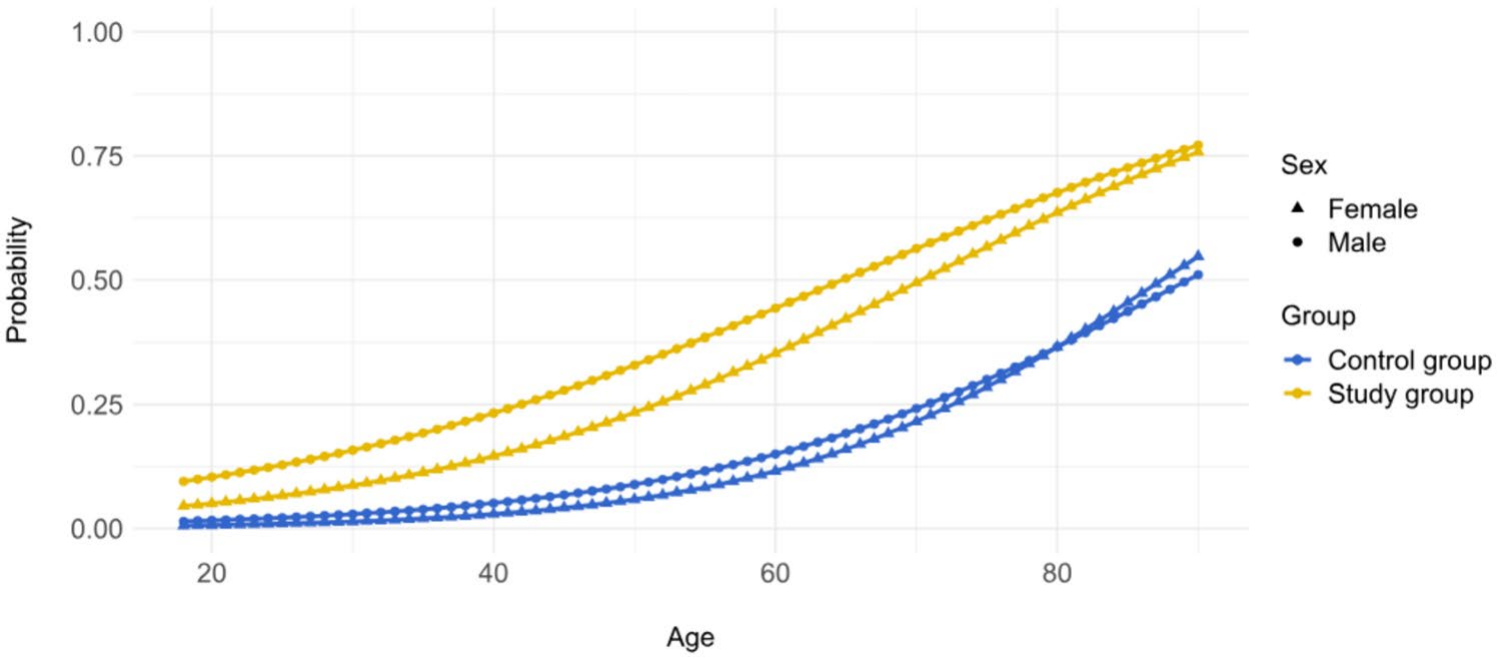
Ten-year age- and sex-specific probability of MR-related complications in the study group vs. control group

Age- and sex-specific prevalence of MR-related complications was calculated in the study group vs. control group (Fig. 3). The risk of experiencing at least one MR-related complication in a 65-year old man with an asymptomatic MR during a 10-year interval was 50%; more than double as compared to an age-matched male patient without MR.

Male patients in the study group had a significantly higher risk of MR-related complications as compared to female patients in all age groups (OR: 2.65, 95%CI: 0.754–1.193, P < 0.0001) (Fig. 3). In contrary, there was no difference in the occurrence of MR-related complications between male and female patients in the control group (Fig. 3).

Finally, age- and sex-specific probabilities of developing specific MR-related complications during a 10-year follow-up were analyzed in the study group vs. control group (Supplemental Figures S4A-D). The age- and sex-specific differences between the study and the control group were most obvious for the risk of developing new congestive heart failure (Figure S4A) and new-onset atrial fibrillation (Figure S4B).

### Ten-year survival in the entire study group

**Fig. 4:**
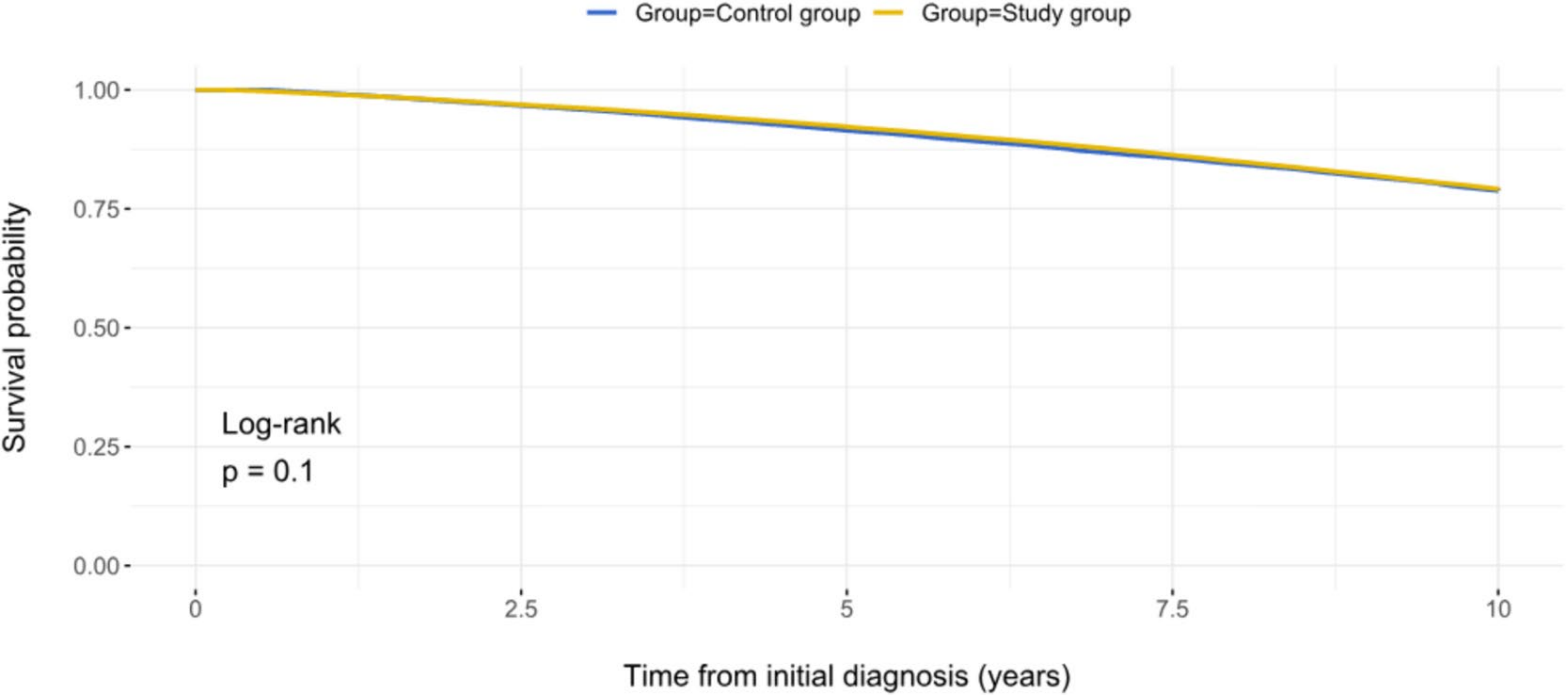
Ten-year survival in the entire study group (i.e., with and without MR-related complications) vs. control group

There was no significant difference in the 10-year survival between the entire study group (i.e., including patients with and without MR-related complications during the follow-up) and the control group (Fig. 4). A total of 11,467 (20.3%) patients died in the study group vs. 11,552 (20.4%) in the control group (OR: 0.991, 95%CI: 0.0022–0.0024, P = 0.535).

Due to higher prevalence of MR-related complications in male vs. female patients in the study group ***(s. ***Figure 1***)***, there has been a sex-specific survival difference between the study group and the control group. A total of 4714 (25.6%) male patients died in the study group vs. 4468 (24.3%) in the control group (OR: 1.07, 95%CI: 0.004–0.022, P = 0.003). On the contrary, 6753 (17.7%) female patients died in the study group as compared to 7084 (18.6%) in the control cohort (OR: 0.94, 95%CI: -0.014- -0.003, P = 0.002) (Supplemental Figures S2A-B).

### Ten-year survival in the study group with vs. without MR-related complications

**Fig. 5:**
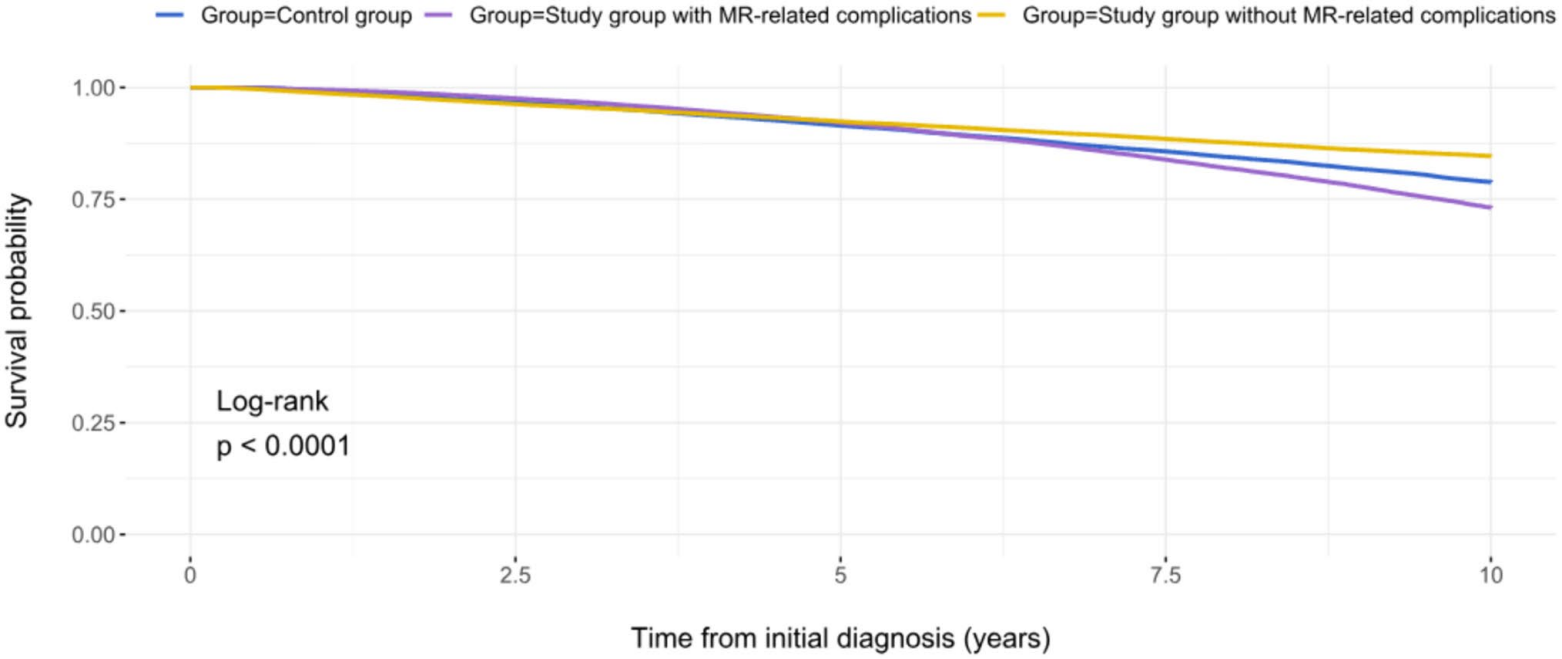
Ten-year survival in the study group with vs. without MR-related complications (at least one) vs. control group

In the next step, we analyzed the survival in the study group separately for the patients who experienced at least one MR-related complication (i.e., study group with MR-related complication) vs. those who remained asymptomatic during a 10-year follow-up (Fig. 5). Patients in the study group who experienced at least one MR-related complication had a significantly worse 10-year survival as compared to the control group (P_log-rank_ < 0.0001). The occurrence of at least one MR-related complication during the 10-year follow-up was associated with a significantly worse 10-year survival (HR: 1.802, 95%CI: 1.736–1.871, p < 0.0001).

### The impact of specific MR-related complications on 10-year survival

Further analyses were performed to evaluate the impact of specific MR-related complications (i.e., congestive heart failure, atrial fibrillation, pulmonary hypertension, and cardiac decompensation) on 10-year survival (Supplemental Figures S3A-D). The occurrence of every single MR-related complication in the study group was associated with a significantly worse 10-year survival (Supplemental Figures S3A-D). The most important prognostic impact was demonstrated for the new onset of congestive heart failure (Supplemental Figure S3A). Adjusted for age and sex, Cox proportional hazard regression revealed a significantly increased 10-year mortality rate in individuals who developed the new-onset congestive heart failure (HR: 1.1317, 95% CI: 1.089–1.177, P < 0.0001).

### Secondary outcomes: mitral valve interventions and hospital admission

A total of 1530 (2.7%) patients in the study group underwent mitral valve (MV) intervention during 10-year follow-up vs. 135 (0.2%) individuals in the control group (OR: 11.6, 95%CI 0.023–0.026, P < 0.0001). Male patients with an asymptomatic MR had MV intervention more frequently as compared to female patients (i.e., 4.4% vs. 1.9%, respectively).There was no difference in MV intervention frequency between male and female individuals in the control group (i.e., 0.3% vs. 0.2%, respectively). Among MV interventions, surgical mitral valve repair including annuloplasty and leaflet repair was the most common procedure (Supplemental Table S4). In the study group, 63 of 1530 patients (4.3%) who underwent MV intervention had more than one hospital admission with a corresponding OPS code for MV intervention during a 10-year follow-up. In the control group, 4 of 131 patients (3.1%) required more than one hospital admission for MV intervention (OR: 1.42, 95%CI: -0.023–0.048, P = 0.652).

## Discussion

The key findings of our current study are summarized in the following bullet points and discussed in detail in the subsequent paragraphs:

- At the time point of MR diagnosis, more than one half of patients are asymptomatic
- Every second patient with an asymptomatic MR experience at least one MR-related complication during a 10-year follow-up
- MR-related complications are associated with a significantly worse 10-year survival in the asymptomatic MR cohort
- The occurrence of MR-related complications in asymptomatic MR patients is age- and sex-dependent

### Prevalence of the asymptomatic MR

No systematic heart check program or screening for valvular heart disease exists in the German health care system. Hence, the prevalence of asymptomatic MR is largely unknown. The only available evidence results from the few population-based studies that included a transthoracic echocardiography as a part of their evaluation protocol (e.g., OxValve, Hamburg City Health Study, Euro Heart Survey) [2, 15, 16]. The issue of asymptomatic MR at the time point of echocardiographic diagnosis has been highlighted by the OxValve Population Cohort study [2]. The authors revealed a major burden of undiagnosed valvular heart disease (VHD) which affected one in two of the elderly population ≥ 65 years, especially in lower socioeconomic classes. Newly diagnosed moderate-to-severe MR was found in 2.3% of this large asymptomatic population with an increasing prevalence in older age groups. The OxValve Population Cohort Study data suggested that the number of individuals in UK aged ≥ 65 years with moderate or severe VHD will double in prevalence in 30 years, while most of them will remain undiagnosed [2].

We followed a different approach to evaluate the incidence of asymptomatic MR which was based on health insurance claims data analysis from Germany’s second largest health insurance fund BARMER which maintains longitudinal data on outpatient and inpatient medical services for approximately 10% of Germany’s population. The BARMER cohort has been extensively validated and used widely for epidemiological research before [8–10, 17, 18]. Out of 90,245 insured individuals with a newly diagnosed MR in the BARMER cohort between 2008 and 2011, 62.7% had no symptoms or secondary complications attributable to MR according to the German Modification of the International Classification of Diseases (ICD-10-GM) and were, therefore, classified as having asymptomatic MR. These findings correlate well with the results of the OxValve study that showed that more than one half of clinically significant VHD remains undetected in the elderly population [2].

### MR-related complications in the asymptomatic MR cohort

There is an obvious shortage of longitudinal data on the asymptomatic MR cohort. The most valuable information on the natural course of asymptomatic MR comes from the data published by Dr. Sarano from the Mayo Clinic [18]. The authors followed prospectively 456 patients with an asymptomatic degenerative MR and reported on 5-year longitudinal follow-up outcomes. The prevalence of MR-related complications (i.e., death from cardiac causes, congestive heart failure, or new atrial fibrillation) was 33% at 5-year follow-up in the whole study cohort and differed significantly according to the severity of MR (i.e., effective regurgitant orifice and the regurgitant volume). Asymptomatic MR patients with a severe MR (i.e., effective regurgitant orifice of at least 40mm^2^) had 62% risk of MR-related complications at 5-year follow-up as compared to 15% risk of MR-related complications among those with a mild MR (i.e., effective regurgitant orifice of less than 20mm^2^). Although our current study does not provide quantitative information on MR severity, MR-related complications rate at 5-year follow-up was 32% in our whole study group and, thereby, very comparable to the findings by the Mayo group [19].

Of note, new congestive heart failure and new-onset atrial fibrillation were the most common MR-related complications in our asymptomatic MR cohort, both occurring at least three-times more frequently as compared to the age- and sex-matched healthy controls.

It is important to mention that the ICD is not sensitive to the cause of MR as it cannot discriminate for functional and degenerative MR. It is therefore possible, that some patients were suffering from asymptomatic heart failure with functional MR at the time of the index diagnosis of MR. Symptoms of heart failure over time may have resulted from worsening heart failure rather than progressive MR.

### MR-related complications and survival in the asymptomatic MR cohort

Our analysis revealed that asymptomatic MR per se is not a predictor of a diminished 10-year survival as compared to age- and sex-adjusted healthy population (Fig. 4). However, this applies only for those patients with an asymptomatic MR who had no MR-related complication during a 10-year follow-up (Fig. 5). The occurrence of at least one MR-related complication was associated with a reduced 10-year survival as compared to the age- and sex-matched control group. New congestive heart failure and new-onset atrial fibrillation were the most prognostically relevant complications, negatively influencing 10-year survival of the asymptomatic MR cohort (Figures S3A-B). Therefore, the definition of baseline characteristics and/or echocardiographic markers that are associated with a higher risk of new congestive heart failure and atrial fibrillation in asymptomatic MR patients could identify the target population for an early MV intervention in an asymptomatic MR stage.

Previous study from the Mayo Clinic compared 5-year survival in asymptomatic degenerative MR patients, stratified by the severity of MR (i.a., according to an effective regurgitant orifice area) [19]. The authors found the lowest survival rate (i.e., 58% at 5-year follow-up) among the asymptomatic MR patients who had an effective regurgitant orifice area ≥ 40mm^2^. On the other hand, asymptomatic MR patients with an effective regurgitant orifice area < 20mm^2^ had the highest 5-year survival that was comparable to a general population. Another study on the prognostic impact of MR-related complications / secondary outcome markers on the post-surgical survival of degenerative MR patients was published by Dr. Butcher et al. from the MIDA (Mitral Regurgitation International Database) registry [6]. The authors found that the presence of secondary outcome determinants (i.e., left atrial volume index ≥ 60ml/m^2^, atrial fibrillation, pulmonary artery systolic pressure ≥ 50 mmHg and ≥ moderate tricuspid regurgitation) in patients with severe degenerative MR were independently associated with a worse post-surgical survival. Furthermore, the increasing number of outcome determinants was associated with an excess post-surgical mortality (i.e., HR 2.6, 95% CI 1.7 – 3.8, P < 0.001 in the presence of three or four outcome determinants). The authors concluded that patients with severe degenerative MR and phenotype characterized by an increasing number of secondary outcome determinants should be considered for earlier surgery.

### Impact of age and sex on the occurrence of MR-related complications

The impact of age on the occurrence of MR-related complications and survival in patients with asymptomatic MR had been already demonstrated in the previous studies [3, 19]. Similarly, our results demonstrate a linearly increasing risk of MR-related complications with an advancing age, both in male and in female patients with an asymptomatic MR (Fig. 3).

Furthermore, we found an increased risk of MR-related complications in male vs. female patients with an asymptomatic MR, which was a constant finding in all age groups (Fig. 3). This sex-specific difference was most obvious in the prevalence of new congestive heart failure (Figure S4A) and new-onset atrial fibrillation (Figure S4B). This finding may potentially explain the significantly worse 10 year survival of male patients with an asymptomatic MR as compared to healthy controls (Figure S2A). To the contrary, no difference in the occurrence of these complications was found between male and female individuals in the control group.

A valid explanation for an increased risk of MR-related complications in male patients with an asymptomatic MR is lacking. This issue has not been addressed in the previous population-based studies of asymptomatic MR patients. Our findings (i.e., significantly worse 10-year survival in male patients with an asymptomatic MR) are somehow contradictory in the context of previously published data on sex-specific outcomes of the surgically treated MR patients [20, 21]. Because female patients are referred later to surgery (i.e., older age and higher prevalence of secondary outcome markers/ comorbidities), their surgical risk is higher and perioperative outcomes worse, as compared to the male counterparts [20, 21]. However, these studies reflect predominantly the referral patterns of tertiary care institutions and do not necessarily apply to population-based cohorts. Therefore, sex-related differences in the MR presentation should be in-depth analyzed in the ongoing population-based studies.

### Mitral valve interventions

Despite high occurrence rate of MR-related complications in the asymptomatic MR cohort (i.e., 50% during 10-year follow-up), MV intervention was performed in only 2.7% patients in the study group. This finding seems somehow counterintuitive, considering the significantly worse 10-year survival in asymptomatic MR patients who had MR-related complications. On the other hand, asymptomatic MR patients without MR-related complications had a 10-year survival that was comparable to the age- and sex-matched general population.

Therefore, the identification of specific phenotypes in the asymptomatic MR patients that indicate MR-related complications should be a future focus of research. Such patients could be candidates for early surgery/intervention to prevent the occurrence of MR-related complications which would potentially translate into improved survival. Similar efforts have been already made in the setting of tertiary care centers by establishing the MIDA-Q score for mortality prediction in degenerative MR [22]. A comparable scoring system is necessary in the population-based setting to reliably stratify asymptomatic MR patients in future screening programs.

## Implications for the screening strategy

The extrapolation of the data from this study (i.e., 8.7 million insured German residents) to the population in Germany (i.e., 85 million in 2024) provides a rough estimate for the potential benefits of a nation-wide screening program for mitral valve heart disease. Considering ca. 1% prevalence of newly diagnosed MR in the BARMER insured population, approximately 850.000 individuals with MR could be potentially expected in the German population. Based on this assumption, echocardiographic screening of approximately 100 individuals would be required to potentially identify one subject with MR. By comparison, 350 colonoscopies are needed in the German colorectal cancer screening program to identify one colorectal cancer in the previously unscreened population at age of 55 [23]. The cost-effectiveness of echocardiographic screening could be further increased by definition of age cut-off in the VHD screening programs (e.g. > 50 years) (Fig. 3). Furthermore, an echocardiographic screening would simultaneously capture other forms of VHD, in particular degenerative aortic stenosis which represents the second most common form of VHD in the adult Western population [2].

## Limitations

Our study has some limitations that have to be considered when interpreting the results. Firstly, our analysis is based on retrospective observational data from the health insurance claims of BARMER. Although this fund covers a substantial sample size and provides representative data for the German population, it is important to note that retrospective observational studies are associated with inherent risks of selection biases and unaccounted confounding factors. Routine data, which are collected primarily for billing and administrative purposes, may lack the detailed clinical nuances required for a comprehensive understanding of certain medical conditions. Additionally, the billing data may introduce bias due to the inclusion of individuals utilizing the healthcare system, thus limiting generalizability to the overall population. Secondly, diagnoses were identified using the German Modification of the International Classification of Diseases (ICD-10-GM), and procedures were identified using the Operations and Procedures Codes (OPS). These classification systems are widely used and established, but differences in coding and documentation between medical facilities could introduce inaccuracies. Furthermore, the utilization of administrative data may limit the accessibility of clinical information required for a thorough risk assessment. Thirdly, it should be noted that only associations can be described, and that causality cannot be inferred. Moreover, missing clinical data on valve and heart function, etc., and the fact that the Best Medical Treatment is not well captured (confounder), add to the limitations of our study. Additionally, we utilized propensity score matching to reduce potential biases in the observational data. However, it is important to note that the presence of unmeasured confounding factors or unrecorded variables that may have influenced group assignment cannot be entirely ruled out. While propensity score methods can reduce the impact, they cannot eliminate the effects of unobserved heterogeneity. It is important to consider these limitations when interpreting the study results. Future research efforts should aim to address these constraints and validate the external validity of our findings.

## Conclusions

One half of asymptomatic MR patients experience MR-related complications during a 10-year follow-up that are associated with a significantly worse survival. Further research should focus on the definition of asymptomatic MR patient’s cohorts at increased risk for complications at the population level, to potentially impact our decision-making process regarding early MV surgery/intervention.

## Supplementary Information

Below is the link to the electronic supplementary material.

Supplementary file1 (PDF 741 kb)

## Data Availability

The anonymized source data will be made available on reasonable request.
