# Supplementary material for: Prognostic impact of disease-related complications in asymptomatic mitral regurgitation: a health insurance claims analysis
Source: Clin Res Cardiol. 2024 Aug 28;114(7):856–66. doi: 10.1007/s00392-024-02532-0 (PMC12202618; doi:10.1007/s00392-024-02532-0)

**Supplementary Material**

**Table S1: OPS codes and terms for mitral valve surgery**

| OPS code | OPS term |
| --- | --- |
| 5-351.1 | Replacement of the mitral valve, open surgery |
| 5-351.2 | Mitral valve replacement, thoracoscopic |
| 5-353.1 | Mitral valve, annuloplasty |
| 5-353.2 | Mitral valve, leaflet reconstruction |
| 5-35a.3 | Implantation of a mitral valve replacement |
| 5-35a.4 | Minimally invasive mitral valve reconstruction |

German Institute for Medical Documentation and Information. 2023. "Classifications - OPS (Operationen- und Prozedurenschlüssel).", https://www.dimdi.de/static/de/klassifikationen/ops/kode-suche/opshtml2023/.

**Table S2: Patients' characteristics before and after Propensity Score Matching in each cohort**

| *Before Propensity Score Matching* | | |
| --- | --- | --- |
| Variable | Study group | Control group |
| Age, years | 68 (58 to 74) | 49 (35 to 61) |
| Male | 18389 (33) | 1589299 (36) |
| Female | 38188 (67) | 2843428 (64) |
| *After Propensity Score Matching* | | |
| Variable | Study group | Control group |
| Age, years | 68 (58 to 74) | 68 (58 to 74) |
| Male | 18389 (33) | 18389 (33) |
| Female | 38188 (67) | 38188 (67) |

Data presented as median (25th-75th percentile), or n (%)

**Table S3: Most common documented comorbidities in the study vs control groups**

| ICD code | ICD term | Study group | Control group |
|---|---|---|---|
| I10 | Essential (primary) hypertension | 41475 (73) | 28571 (50) |
| E78 | Disorders of lipoprotein metabolism and other lipidemias | 29961 (53) | 19566 (35) |
| M54 | Back pain | 25266 (45) | 19548 (35) |
| H52 | Accommodation and refraction disorders | 24367 (43) | 19061 (34) |
| N95 | Climacteric disorders | 14013 (25) | 11034 (20) |
| M47 | Spondylosis | 12467 (22) | 8398 (15) |
| M17 | Gonarthrosis (Arthrosis of the knee joint) | 10453 (18) | 8219 (15) |
| F32 | Depressive episode | 10558 (19) | 8038 (14) |
| E11 | Non-insulin-dependent diabetes mellitus (Type 2 diabetes) | 8881 (16) | 7662 (14) |
| E04 | Other non-toxic goiter | 11753 (21) | 7662 (14) |
| I83 | Varicose veins of the lower extremities | 10049 (18) | 7252 (13) |
| M53 | Other diseases of the spine and back, not elsewhere classified | 10463 (18) | 7085 (13) |
| F45 | Somatoform disorders | 10503 (19) | 6851 (12) |
| H35 | Other affections of the retina | 9145 (16) | 6635 (12) |
| E66 | Obesity | 8657 (15) | 5750 (10) |

Data presented as n (%). The proportion of patients with the most frequently documented ICD codes from the year of initial diagnosis of mitral valve disease (outpatient data) is presented

**Table S4: Patients undergoing mitral valve intervention procedures in the asymptomatic MR cohort vs control group in the ten-year follow-up**

| OPS code | OPS term | Study group | Control group |
|---|---|---|---|
| 5-3511 | Replacement of the mitral valve, open surgery | 363 (0.6) | 36 (0.1) |
| 5-3512 | Mitral valve replacement, thoracoscopic | 36 (0.1) | 2 (0.0) |
| 5-3531 | Mitral valve, annuloplasty | 881 (1.6) | 50 (0.1) |
| 5-3532 | Mitral valve, leaflet reconstruction | 562 (1.0) | 26 (0.0) |
| 5-35a3 | Implantation of a mitral valve replacement | 11 (0.0) | 1 (0.0) |
| 5-35a4 | Minimally invasive mitral valve reconstruction | 272 (0.5) | 49 (0.1) |

Data presented as n (%)

**Figure S1A: Prevalence of congestive heart failure in the asymptomatic MR vs control groups**

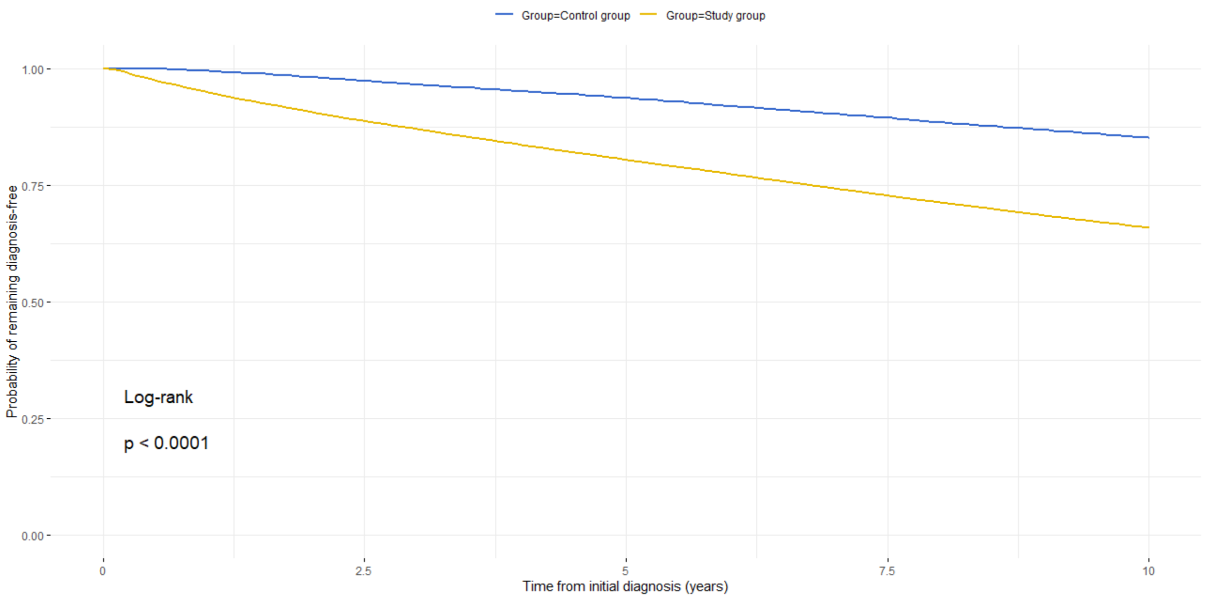


**Figure S1B: Prevalence of new onset atrial fibrillation in the asymptomatic MR vs control groups**

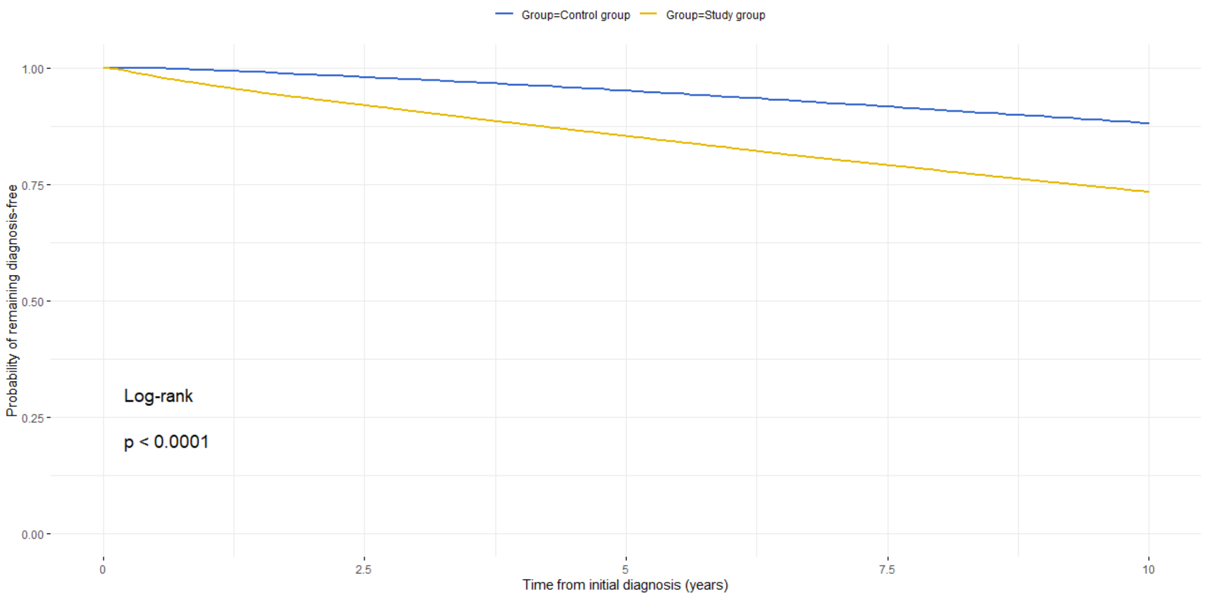

## Figure S1C: Prevalence of pulmonary hypertension in the asymptomatic MR vs control groups

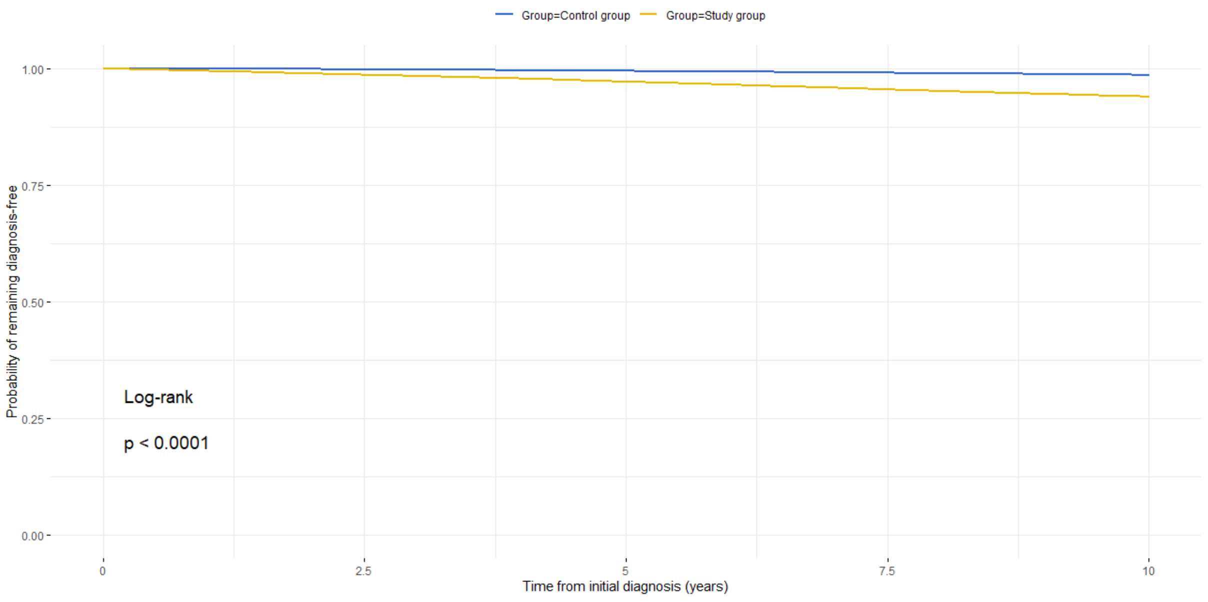


## Figure S1D: Prevalence of cardiac decompensation in the asymptomatic MR vs control groups

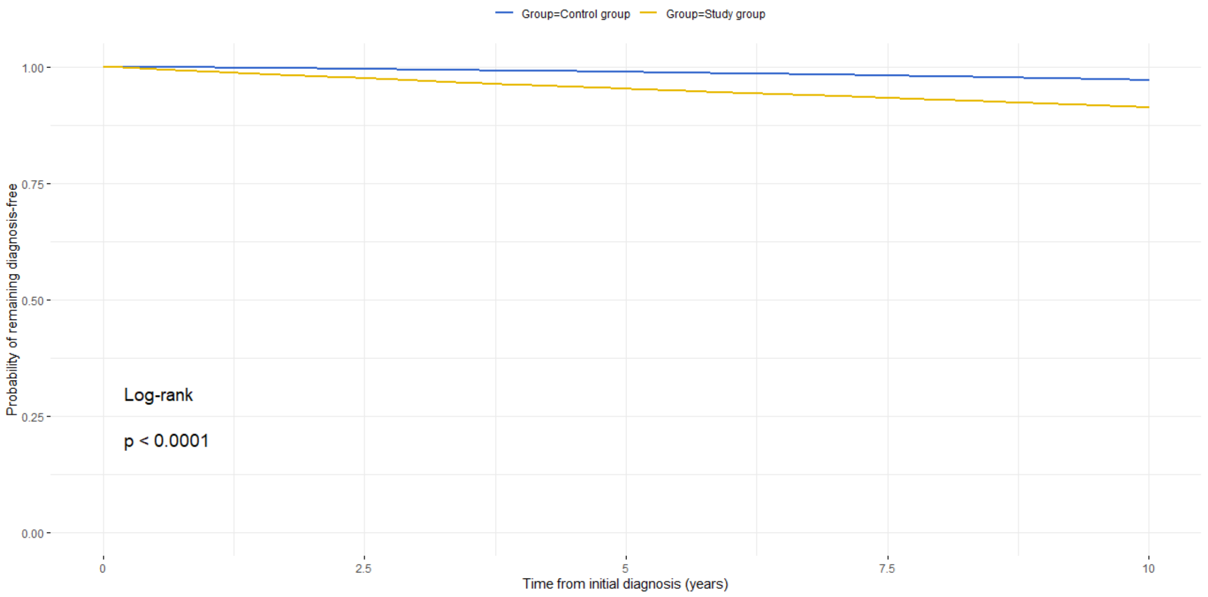

**Figure S2A: Kaplan-Meier survival curves: Female patients**

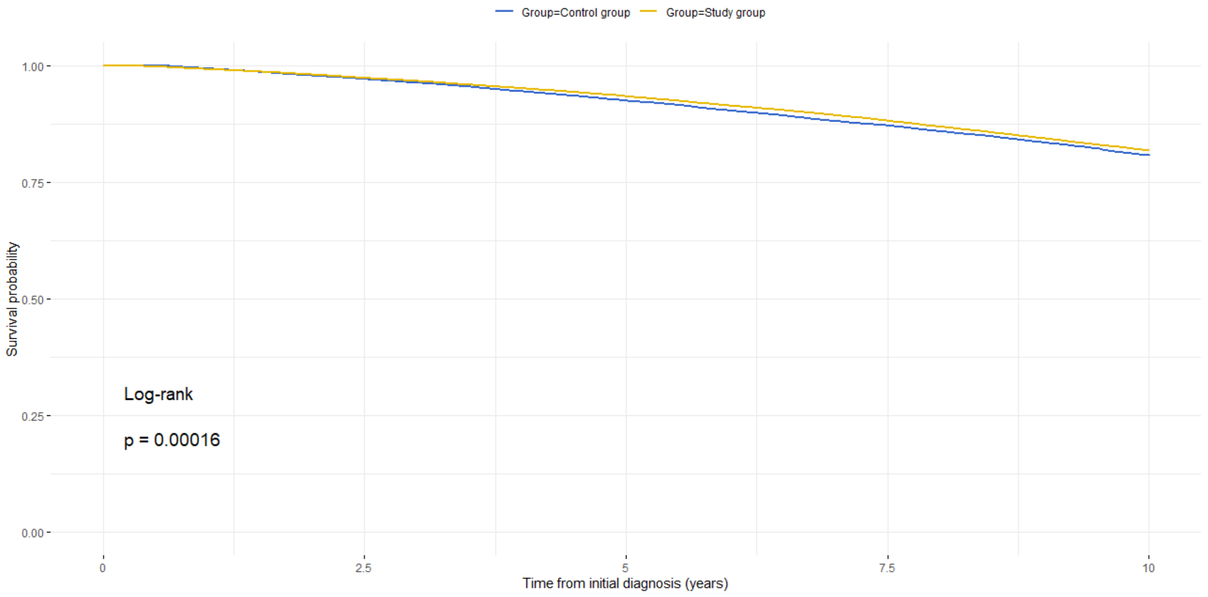


**Figure S2B: Kaplan-Meier survival curves: Male patients**

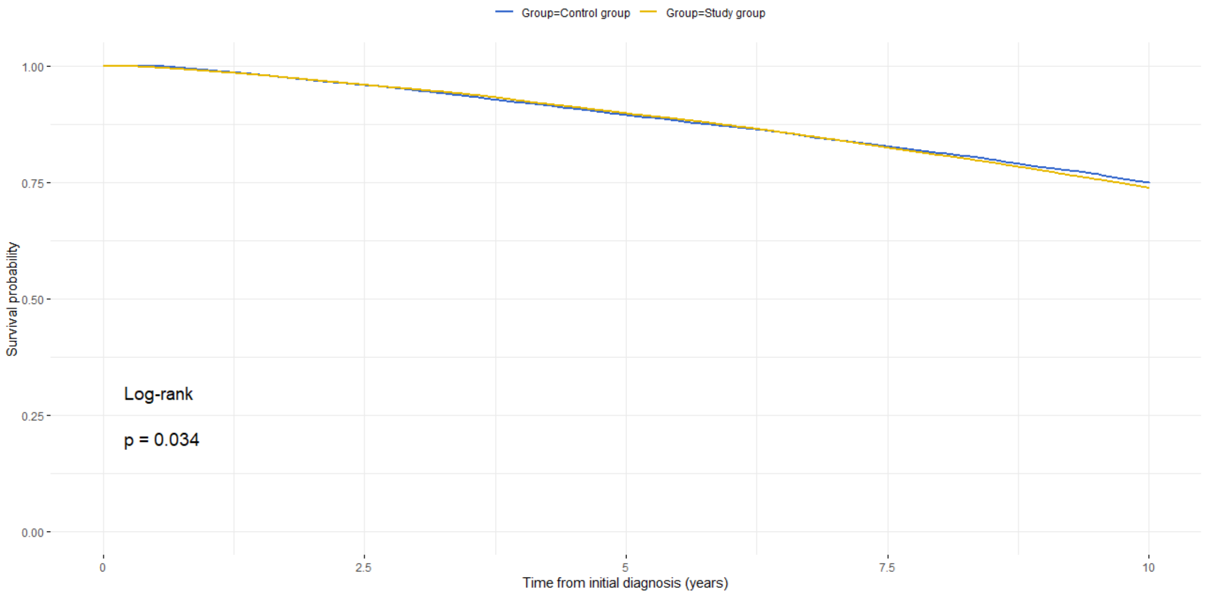

**Figure S3A: Ten-year survival in asymptomatic MR patients with new-onset congestive heart failure vs without congestive heart failure vs control group**

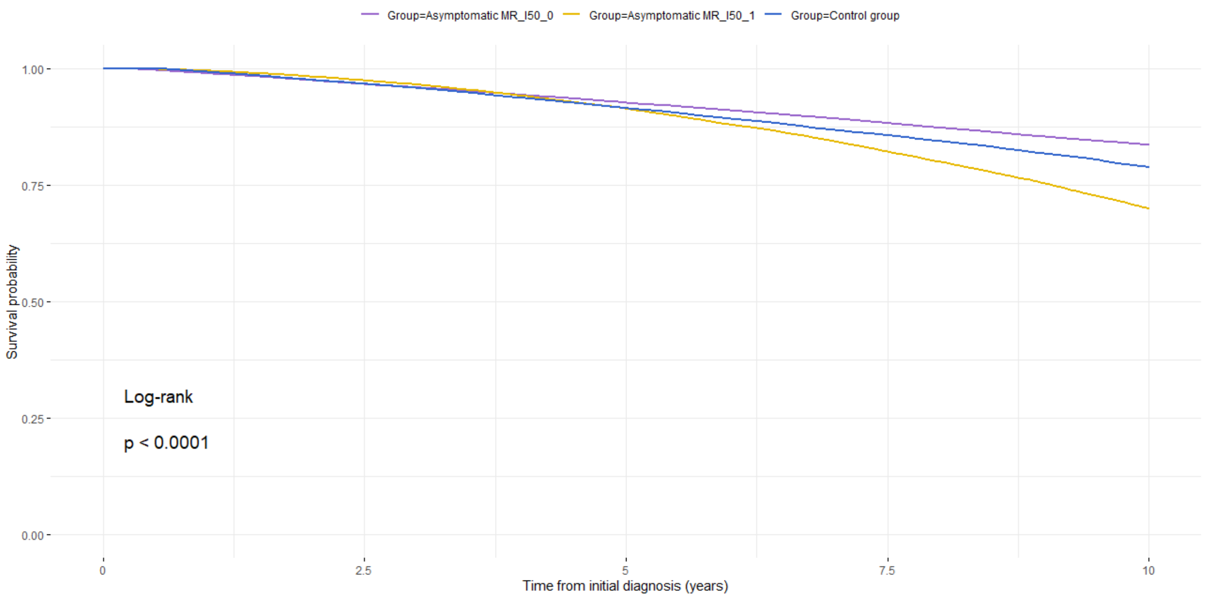


Asymptomatic MR_I50_0 = patients without new onset congestive heart failure, Asymptomatic MR_I50_1 = patients with new-onset congestive heart failure

**Figure S3B: Ten-year survival in asymptomatic MR patients with new-onset atrial fibrillation vs without atrial fibrillation vs control group**

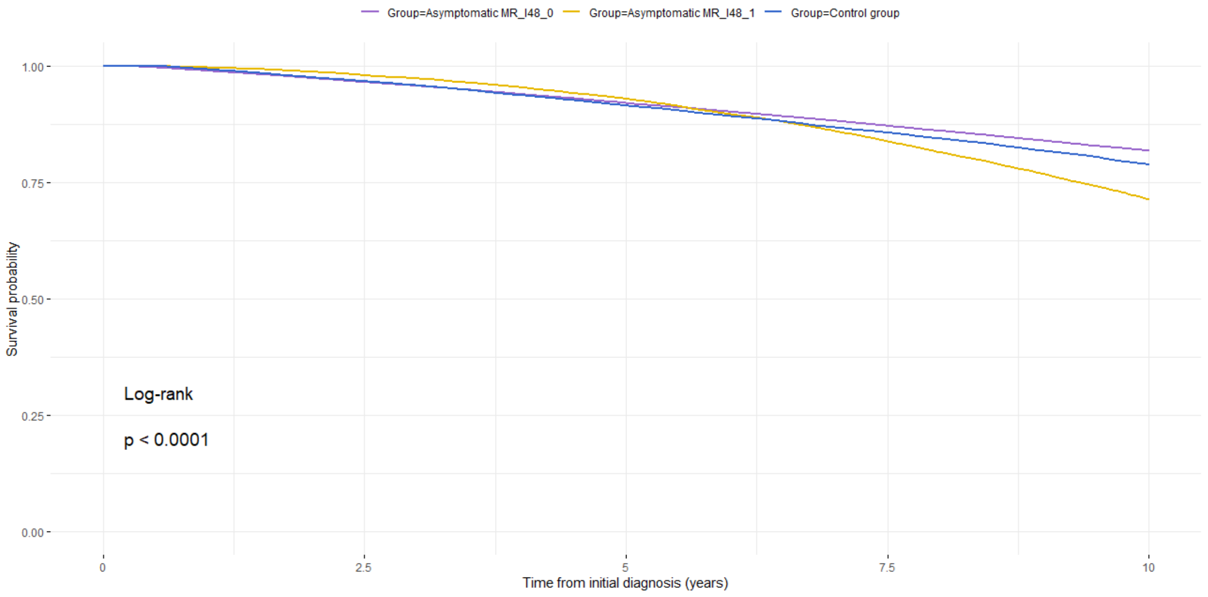


Asymptomatic MR_I48_0 = patients without new onset atrial fibrillation, Asymptomatic MR_I48_1 = patients with new-onset atrial fibrillation

**Figure S3C: Ten-year survival in asymptomatic MR patients with new-onset pulmonary hypertension vs without pulmonary hypertension vs control group**

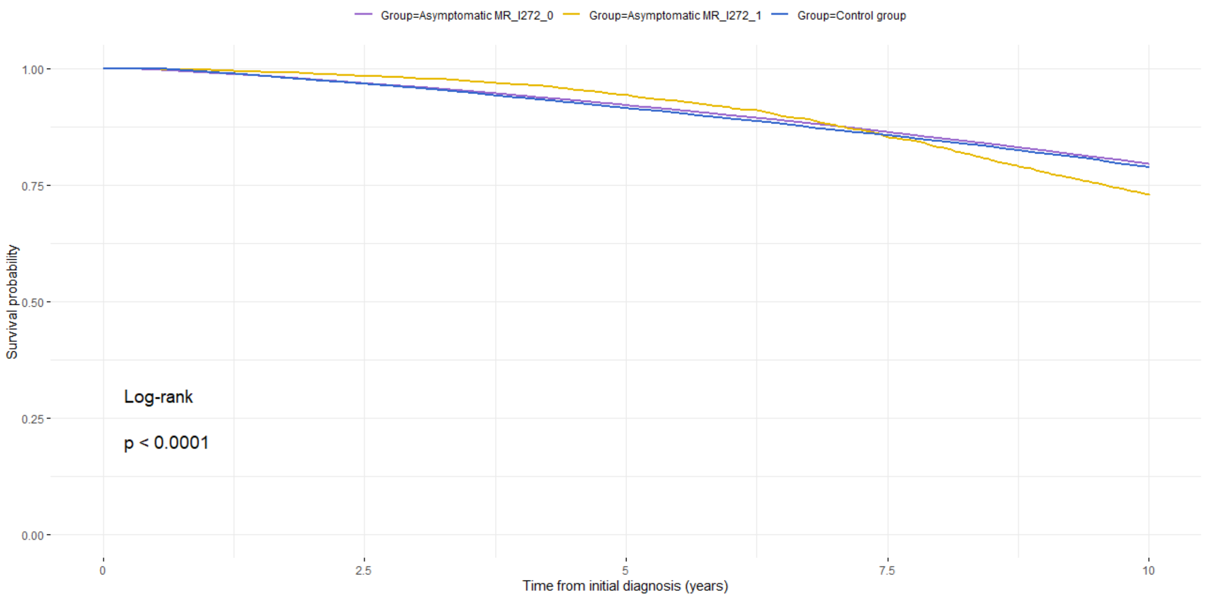


Asymptomatic MR_I272_0 = patients without new onset pulmonary hypertension, Asymptomatic MR_I272_1 = Patients with new onset pulmonary hypertension

**Figure S3D: Ten-year survival in asymptomatic MR patients with new-onset cardiac decompensation vs without cardiac decompensation vs control group**

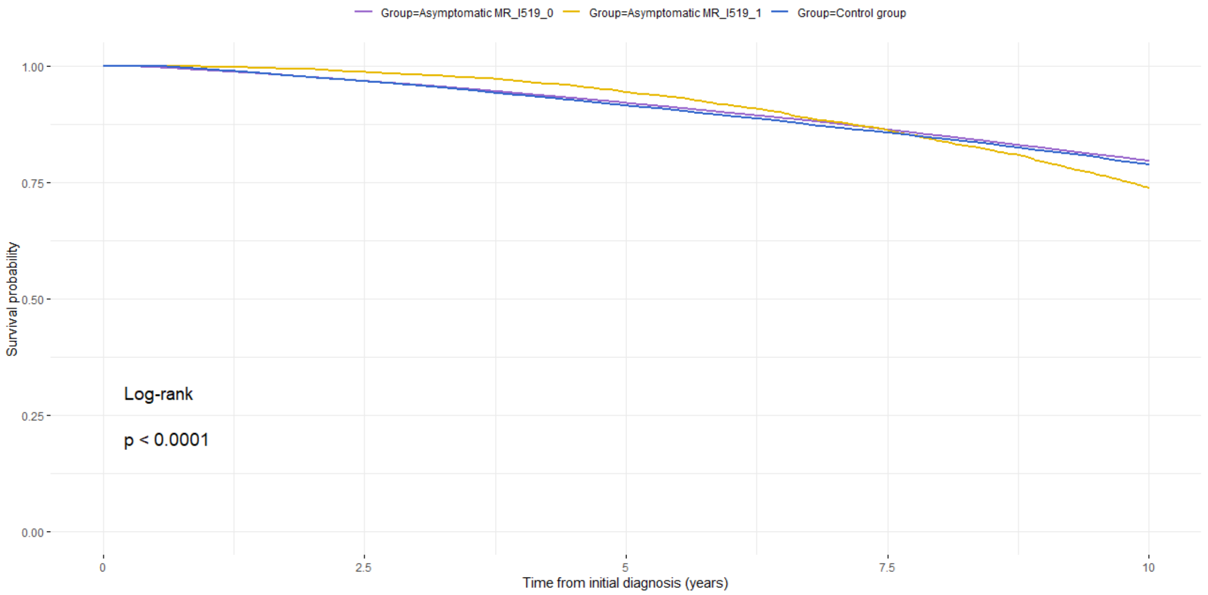


Asymptomatic MR_I519_0 = Patients without new onset cardiac decompensation, Asymptomatic MR_I519_1 = Patients with new onset cardiac decompensation

**Figure S4A: Probability of congestive heart failure during ten-year follow-up per age, sex, and group**

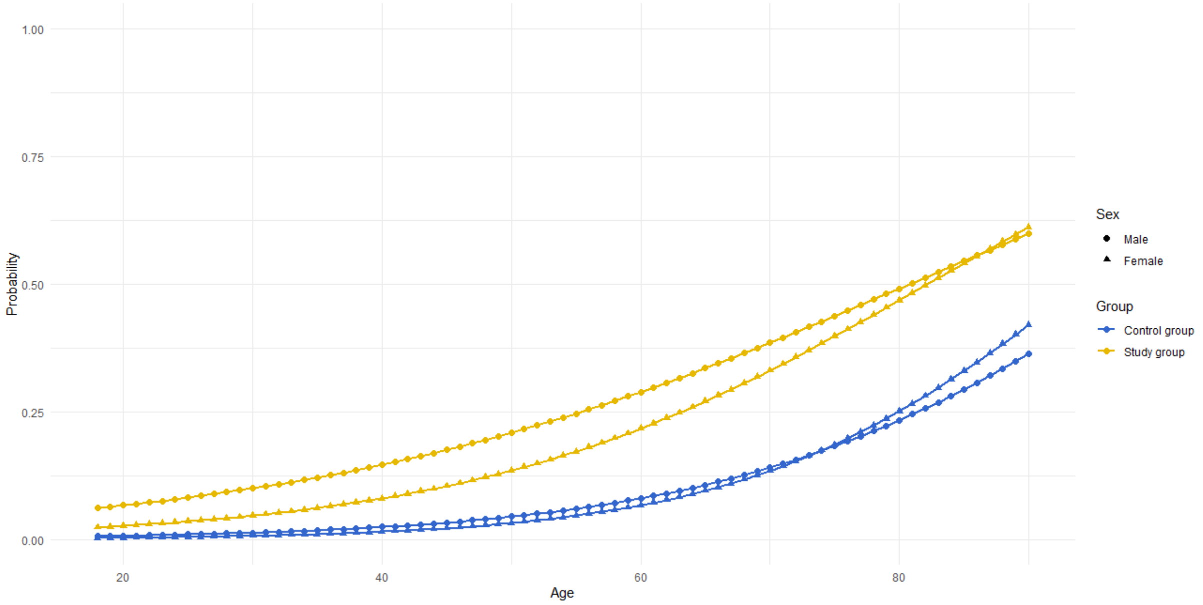


**Figure S4B: Probability of atrial fibrillation during ten-year follow-up per age, sex, and group**

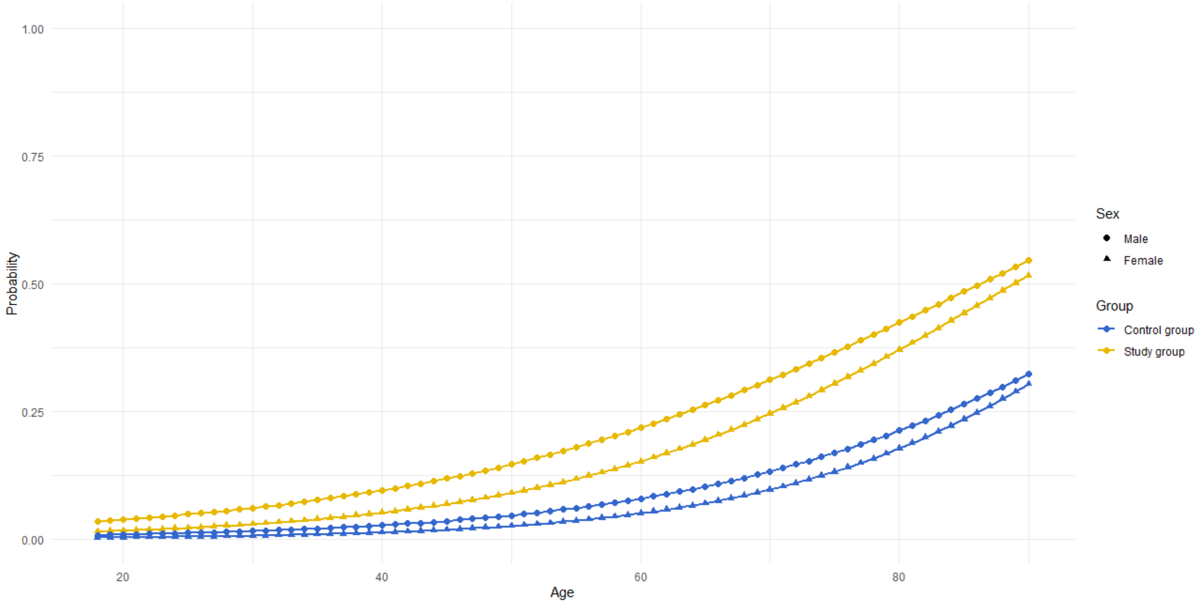

**Figure S4C: Probability of pulmonary hypertension during ten-year follow-up per age, sex, and group**

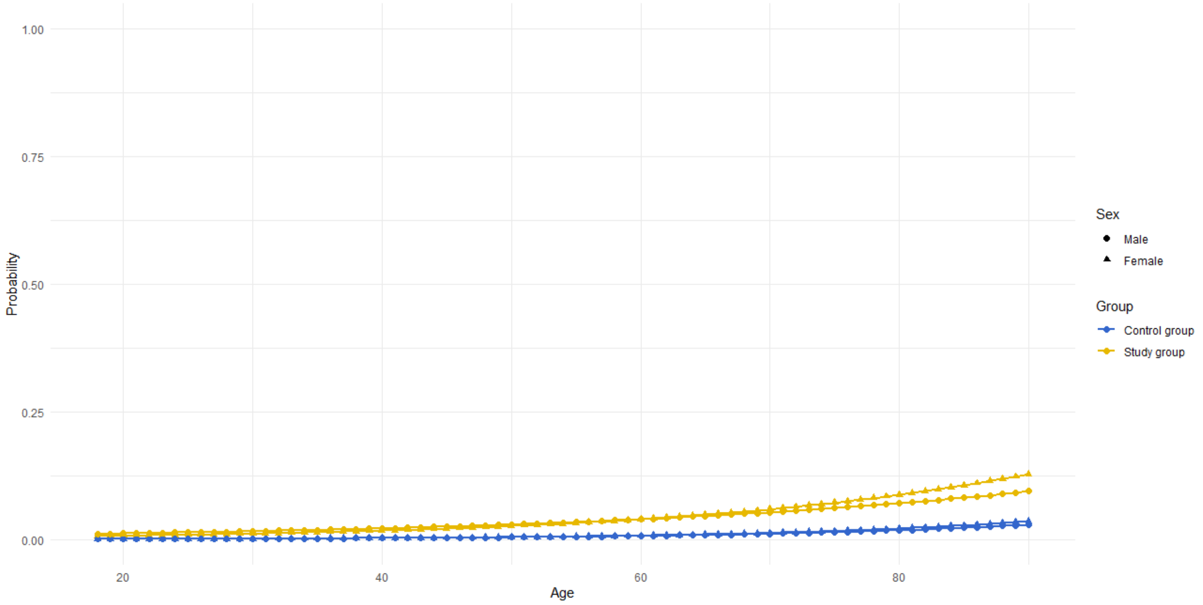


**Figure S4D: Probability of cardiac decompensation during ten-year follow-up per age, sex, and group**

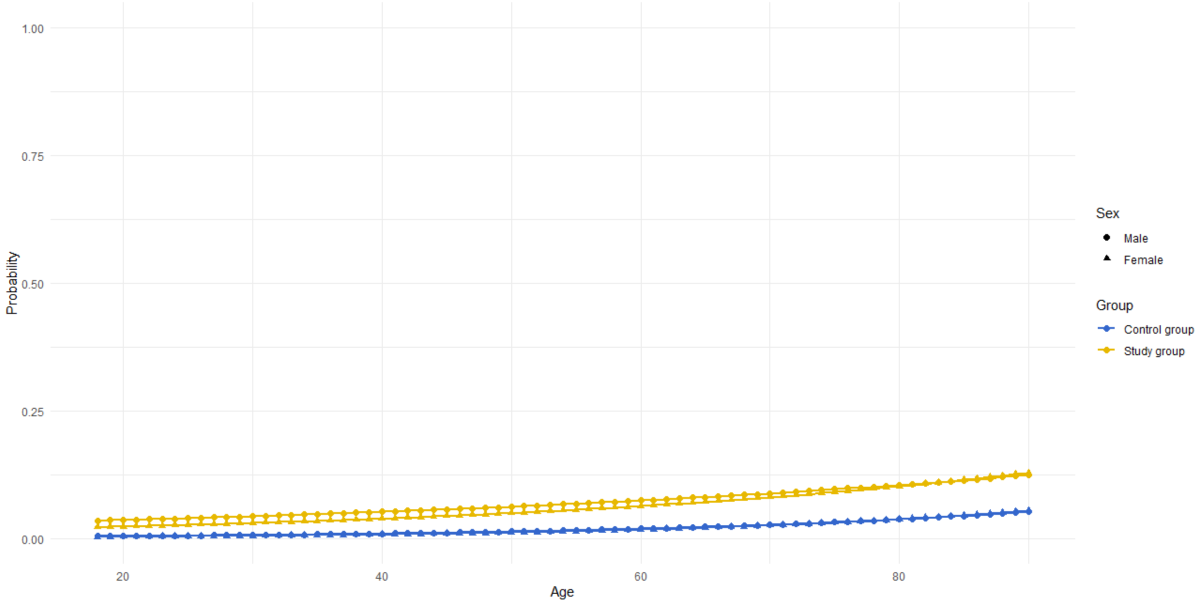

Supplement: Supplementary file 1 — Supplementary file1 (PDF 741 kb) [file 392_2024_2532_MOESM1_ESM.pdf]
